# Characterization of Three Tetrabromobisphenol-S Derivatives in Mollusks from Chinese Bohai Sea: A Strategy for Novel Brominated Contaminants Identification

**DOI:** 10.1038/srep11741

**Published:** 2015-07-01

**Authors:** Ai-feng Liu, Yong Tian, Nuo-ya Yin, Miao Yu, Guang-bo Qu, Jian-bo Shi, Yu-guo Du, Gui-bin Jiang

**Affiliations:** 1 State Key Laboratory of Environmental Chemistry and Ecotoxicology, Research Center for Eco-Environmental Sciences, Chinese Academy of Sciences, P.O. Box 2871, Beijing 100085, China

## Abstract

Identification of novel brominated contaminants in the environment, especially the derivatives and byproducts of brominated flame retardants (BFRs), has become a wide concern because of their adverse effects on human health. Herein, we qualitatively and quantitatively identified three byproducts of tetrabromobisphenol-S bis(2,3-dibromopropyl ether) (TBBPS-BDBPE), including TBBPS mono(allyl ether) (TBBPS-MAE), TBBPS mono(2-bromoallyl ether) (TBBPS-MBAE) and TBBPS mono(2,3-dibromopropyl ether) (TBBPS-MDBPE) as novel brominated contaminants. Meanwhile, the mass spectra and analytical method for determination of TBBPS-BDBPE byproducts were presented for the first time. The detectable concentrations (dry weight) of TBBPS-MAE, TBBPS-MBAE and TBBPS-MDBPE were in the ranges 28–394 μg/g in technical TBBPS-BDBPE and 0.1–4.1 ng/g in mollusks collected from the Chinese Bohai Sea. The detection frequencies in mollusk samples were 5%, 39%, 95% for TBBPS-MAE, TBBPS-MBAE and TBBPS-MDBPE, respectively, indicating their prevailing in the environment. The results showed that they could be co-produced and leaked into the environment with production process, and might be more bioaccumulative and toxic than TBBPS-BDBPE. Therefore, the production and use of TBBPS derivatives lead to unexpected contamination to the surrounding environment. This study also provided an effective approach for identification of novel contaminants in the environment with synthesized standards and Orbitrap high resolution mass spectrometry.

Recently, increasing studies have been carried out to identify novel brominated contaminants in the environment, especially the derivatives, byproducts and degradation products of brominated flame retardants (BFRs)^1^,^2^,^3^. For example, the mono-modified byproducts or degradation products of tetrabromobisphenol-A (TBBPA) derivatives, including TBBPA mono(allyl ether) (TBBPA-MAE), TBBPA mono(2,3-dibromopropyl ether) (TBBPA-MDBPE), have been found in various environment samples, such as soil, sediment, earthworm and mollusks^2^,^3^. More importantly, these byproducts and degradation products showed higher bioaccumulation and toxicity than main technical TBBPA products^2^,^4^,^5^. Due to the similar structures and production process^6^,^7^, there should be more mono-modified byproducts of TBBPA derivatives co-produced and leaked into environment, which could cause widespread contamination and deserve our more attention.

As important alternatives of TBBPA, the most widely used BFR, tetrabromobisphenol-S (TBBPS) and TBBPS bis(2,3-dibromopropyl ether) (TBBPS-BDBPE) are extensively produced and applied in electronic devices, plastics, rubber and textiles^8^. As a result, TBBPS and TBBPS-BDBPE have been detected in waste water at a concentration up to 10 μg/L^9^. TBBPS-BDBPE was also found in herring gull eggs collected from colonies in the Laurentian Great Lakes^10^. Because TBBPS-BDBPE is synthesized by modification of the two phenol groups of TBBPS^7^, the mono-modified byproducts of TBBPS-BDBPE might also be co-produced with technical products and leaked into environment as potential contaminants, such as TBBPS mono(allyl ether) (TBBPS-MAE), TBBPS mono(2-bromoallyl ether) (TBBPS-MBAE) and TBBPS mono(2,3-dibromopropyl ether) (TBBPS-MDBPE). However, the byproducts of TBBPS derivatives were largely ignored in most studies, and there are even no pure standards available. Much still remains unknown about their environmental distribution and risks.

The lack of analytical methods is another main obstacle for identifying novel contaminants. Because of the thermolability of TBBPA, TBBPS and their derivatives, gas chromatography mass spectrometry (GC-MS) is not applicable for direct analysis of these compounds^11^,^12^. The high performance liquid chromatography coupled with tandem MS (HPLC-MS/MS) has been developed for analysis of TBBPA and TBBPS derivatives^3^,^8^,^10^,^13^. However, electrospray ionization (ESI) source was reported with poor sensitivity because of the weak polarity of TBBPA and TBBPS derivatives^8^. Although atmospheric pressure chemical ionization (APCI) and atmospheric pressure photoionization (APPI) mass spectrometry methods have been developed, they were not sufficient for the trace level determination of these derivatives in the environment matrices^10^,^13^. With the rapid development of high resolution mass spectrometry (HRMS), such as time of flight (TOF) HRMS and Orbitrap HRMS, the novel contaminants could be identified and quantified through the full scan acquisition spectrum^14^,^15^,^16^,^17^. An attractive advantage of full scan HRMS is that there is no number limitation of analytes in one single injection, which is enormously beneficial to the retrospective analysis of untargeted contaminants^18^. Furthermore, the exact mass information is helpful for identification of compounds without standards, which largely extends its application^19^. In this view, the combination of ultra HPLC (UHPLC) with Orbitrap Fusion HRMS technique would provide a high accuracy as well as a low detection limit for the mono-modified byproducts of TBBPS-BDBPE.

The aim of this study was to identify three potential byproducts of TBBPS-BDBPE as novel brominated contaminants. The standards of TBBPS-MAE, TBBPS-MBAE and TBBPS-MDBPE were synthesized with high purity. A sensitive and accurate method for simultaneous determination of these novel TBBPS derivatives was developed with UHPLC-Orbitrap HRMS. Their distribution in mollusk samples collected from the Chinese Bohai Sea and potential risks were discussed in detail. The strategy used in this work could also be an effective approach for identifying other novel brominated pollutants related to BFRs.

## Results

### Structure Confirmation of Synthesized TBBPS-MAE, TBBPS-MBAE and TBBPS-MDBPE

The standards of TBBPS-MAE, TBBPS-MBAE and TBBPS-MDBPE were self-synthesized with purities of 99%, 98% and 96%, respectively. The synthesis schemes and ^1^HNMR spectra of these compounds are provided in Figure S1 (Supporting Information).

**Figure 1:**
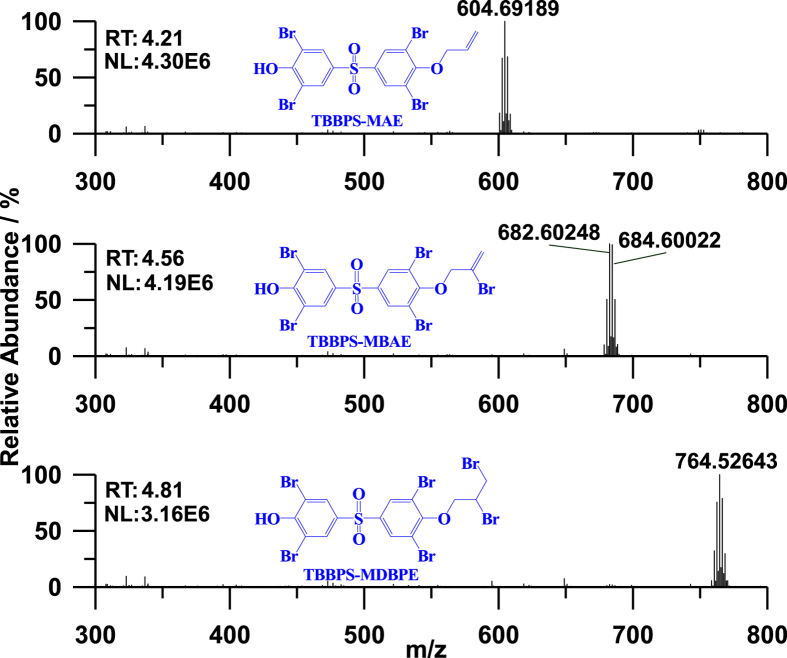
The HRMS spectra of TBBPS-MAE, TBBPS-MBAE and TBBPS-MDBPE (2.5 ng, ESI full scan 100–1000).

**Figure 2:**
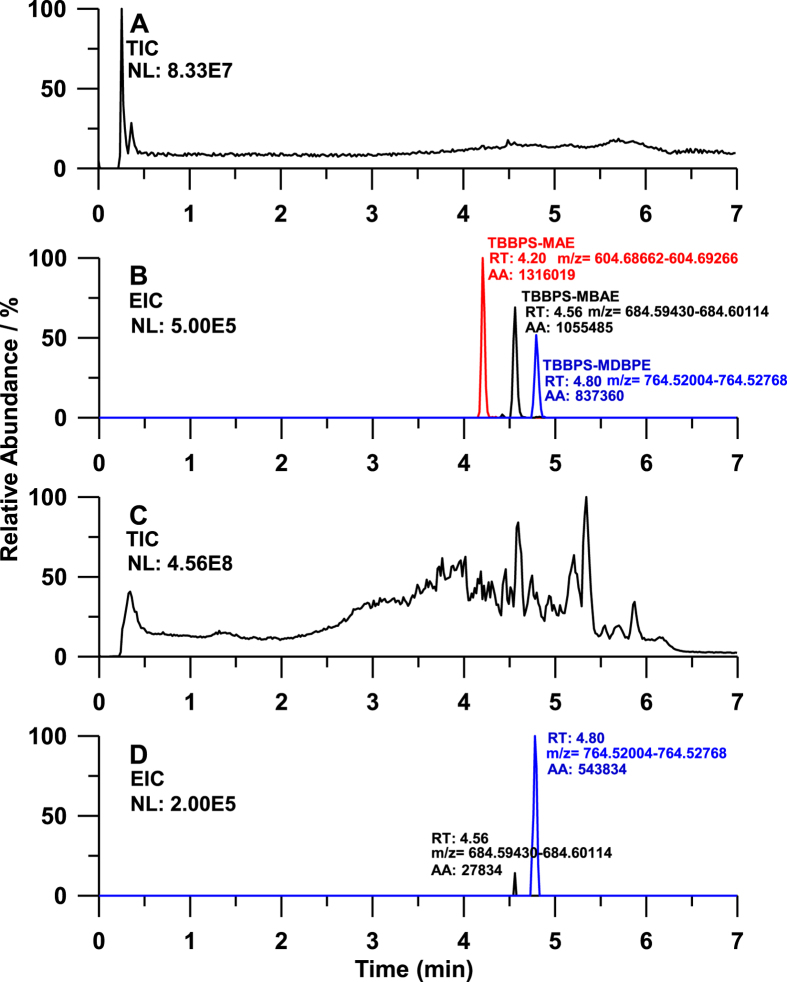
The mass chromatogram of standards and the mollusk sample contained target compounds. **A** and **B**, standards, 10 pg; **C** and **D**, RapS sample of 2010; TIC, total ion chromatogram; EIC, extracted ion chromatogram, mass tolerance, 5 ppm; AA, area.

Orbitrap Fusion HRMS was employed to further identify the target compounds in the full scan mode (Table 1, Fig. 1, Fig. 2A,B and Figure S2). The *m/z* values of [M-H]^−^ for TBBPS-MAE, TBBPS-MBAE and TBBPS-MDBPE were 604.69189, 684.60022/682.60248, and 764.52643, respectively (Fig. 1). The m/z values of [M-H]^−^ ions of TBBPS-MAE, TBBPS-MBAE and TBBPS-MDBPE calculated with Xcalibur software were 604.69196, 684.60043/682.60247 and 764.52659, respectively. The mass errors for TBBPS-MAE, TBBPS-MBAE and TBBPS-MDBPE were all less than 0.4 ppm. The isotope distributions of TBBPS-MAE, TBBPS-MBAE and TBBPS-MDBPE characterized by Orbitrap Fusion HRMS were 1:4:6:4:1, 1:5:10:10:5:1 and 1:6:15:20:15:6:1, respectively. The minimum isotope [M-H]^−^ ions of TBBPS-MAE (600.69635), TBBPS-MBAE (678.60681) and TBBPS-MDBPE (758.53296) were calculated with Orbitrap Xcalibur software to present the molecular composition of [C_15_H_9_Br_4_O_4_S]^−^, [C_15_H_8_Br_5_O_4_S]^−^ and [C_15_H_9_Br_6_O_4_S]^−^, respectively. The precursor ions of TBBPS-MAE (m/z 604.69), TBBPS-MBAE (m/z 682.60) and TBBPS-MDBPE (764.53) generated the same fragment ion clusters at m/z 563.65, 499.69, 484.73, 419.77/417.77, 297.81, 265.84, and 249.85, which were calculated with Xcalibur software to be [C_12_H_4_O_4_Br_4_S]^−^, [C_12_H_4_O_2_Br_4_]^−^, [C_12_H_4_O_4_Br_3_S]^−^, [C_12_H_3_O_2_Br_3_]^−^, [C_6_H_2_O_2_Br_2_S]^−^, [C_6_H_2_O_2_Br_2_]^−^ and [C_6_H_2_OBr_2_]^−^, respectively (Figure S2). The ion clusters at m/z 78.92/80.92 showed the existence of the fragment [Br]^−^. The fragment ion clusters at m/z 508.75/510.75 were only observed in MS^2^ spectrum of TBBPS-MAE, and m/z 522.77/524.77 could only be observed in MS^2^ spectra of TBBPS-MBAE and TBBPS-MDBPE. The fragment ion clusters at m/z 508.75/510.75 and 522.77/524.77 were calculated to be [C_14_H_6_O_4_Br_3_S]^−^ and [C_15_H_8_O_4_Br_3_S]^−^, respectively. The fragment ions were generated through the rearrangement of the molecular and the breakage of ether bond, C-Br bond, C–S bond and S = O bond.

### Method Development and Performance

In the mass chromatogram (Fig. 2), the respective retention times (RTs) of TBBPS-MAE, TBBPS-MBAE and TBBPS-MDBPE were 4.20 min, 4.56 min and 4.80 min. Coupled with Orbitrap Fusion HRMS, the instrument detection limits (IDLs) for TBBPS-MAE, TBBPS-MBAE and TBBPS-MDBPE achieved 0.06 pg, 0.1 pg and 0.1 pg. The IDLs were lower than those detected by HPLC-ESI-MS/MS (Quattro Premier XE, Waters, USA) which were 0.2 pg, 0.5 pg and 0.5 pg for TBBPS-MAE, TBBPS-MBAE and TBBPS-MDBPE, respectively.

The recoveries for TBBPS-MAE, TBBPS-MBAE and TBBPS-MDBPE were all higher than 76% with ultrasound method and accelerated solvent extraction (ASE) method at the spiking amounts of 10 ng in 0.5 g Neverita didyma (Nev) samples (n = 7, Table 2). The recoveries of ultrasound method were slightly higher than ASE method and the standard deviations (SDs) were lower than ASE method. Finally, the samples were extracted by ultrasound method and cleaned by ENVI-carb cartridges. The mean recoveries for TBBPS-MAE, TBBPS-MBAE and TBBPS-MDBPE were all higher than 70% and the SDs were all less than 10% at three different spiking amounts, 100 ng (n = 5), 10 ng (n = 7) and 1 ng (n = 5) (Table 2). The method DLs (MDLs) of TBBPS-MAE, TBBPS-MBAE and TBBPS-MDBPE were 0.04 ng/g dry weight (dw), 0.08 ng/g dw and 0.06 ng/g dw, respectively. The mean recovery of the internal standard, ^13^C labeled 3,5-dibromophenol (ISDBP), was 101% ± 7% at the spiking amount of 10 ng (n = 7). The recoveries of ISDBP from the real samples ranged from 81% to 104% with a mean recovery of 91% and SD of 6% (n = 38). The matrix effects ranged from 0.86 to 1.05 (Table 2) at three different spiking concentrations, 1 ng/mL, 5 ng/mL and 10 ng/mL. The pretreatment method of TBBPS-MAE, TBBPS-MBAE and TBBPS-MDBPE from the real samples was reliable and repeatable. Details for the development and optimization of extraction and solid phase extraction (SPE) cleanup procedure were provided in Supporting Information.

### TBBPS-MAE, TBBPS-MBAE and TBBPS-MDBPE in Technical Products and Mollusk Samples

The technical product of TBBPS-BDBPE purchased from a BFRs factory (purity >90%) was dissolved in methanol at a concentration of 100 μg/mL and determined by Orbitrap Fusion HRMS. The concentrations of TBBPS-MAE, TBBPS-MBAE and TBBPS-MDBPE in this technical TBBPS-BDBPE were 28 μg/g, 87 μg/g and 394 μg/g, respectively.

**Figure 3:**
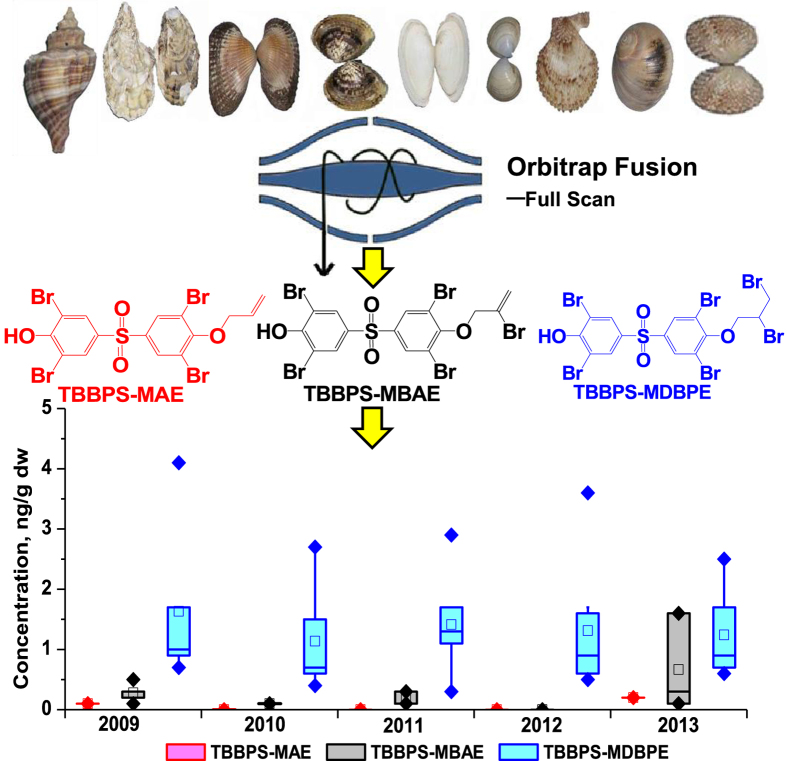
The concentrations of TBBPS-MAE, TBBPS-MBAE and TBBPS-MDBPE in mollusk samples from 2009 to 2013. The concentration of 25^th^ and 75^th^ percentiles is represented by box, the median concentration is represented by middle line. In the figure, “□” represents mean mass concentration value. The whiskers extending from the box are the lowest and highest non-outlier values. “♦” represents the lowest and highest value. The photographs of the mollusks in the figure were taken by Ai-feng Liu.

The concentrations of TBBPS-MAE, TBBPS-MBAE and TBBPS-MDBPE in total 38 mollusk samples collected from Bohai Sea during 2009 to 2013 were also analyzed with an external standard method (Table S1). TBBPS-MAE was only found in two mollusk samples with concentrations of 0.1 ng/g dw and 0.2 ng/g dw. TBBPS-MBAE was detectable in 15 samples with the concentrations ranging from 0.1 to 1.6 ng/g dw. In these 15 samples, thirteen ones had the concentrations between 0.1 and 0.3 ng/g dw. TBBPS-MDBPE was detectable in 36 samples with the concentrations ranging from 0.3 to 4.1 ng/g dw, among which 20 ones contained the compound higher than 1.0 ng/g dw. The detection frequencies of these three compounds were in the order of TBBPS-MDBPE (95%) >TBBPS-MBAE (39%) >TBBPS-MAE (5%). As shown in Fig. 3, the mean concentration of TBBPS-MDBPE was higher than that of TBBPS-MBAE, and the concentration of TBBPS-MAE was the lowest. A typical mass chromatogram for the three compounds detected in mollusk sample is shown in Fig. 2(C,D).

### Environmental Risk Prediction of TBBPS Derivatives

The physical-chemical properties of TBBPS, TBBPS derivatives and other well concerned contaminants were calculated by US EPA EPI Suite V4.1, which has been widely employed for screening of potentially persistent and bioaccumulative contaminants^20^,^21^,^22^,^23^,^24^,^25^,^26^. As shown in Table 3, the log *K*_ow_ values of TBBPS and the derivatives ranged from 5.21 to 9.52, log *K*_oa_ values ranged from 16.83 to 21.83, log *K*_oc_ values ranged from 4.16 to 6.33, and log *K*_aw_ values were all lower than −8.81. The bioconcentration factor (BCF) values of TBBPS-MAE, TBBPS-MBAE and TBBPS-MDBPE were 10730, 13200 and 8829, respectively, which were significantly higher than those of TBBPS (1266), TBBPS bis(allyl ether) (TBBPS-BAE) (4207) and TBBPS-BDBPE (775).

In addition, the potential toxicity of TBBPS-BDBPE, TBBPS-BAE, TBBPS, TBBPS-MAE, TBBPA-MBAE and TBBPS-MDBPE were also estimated with the primary cerebellum granule cells (CGCs) as the model, which were usually used for neurotoxicity studies^13^,^27^,^28^. The IC_50_ of TBBPS, TBBPS-MAE, TBBPA-MBAE and TBBPS-MDBPE were 0.45, 0.19, 0.20 and 0.17 μM, respectively. The IC_50_ of TBBPS-BAE and TBBPS-BDBPE were 13.1 and 11.2 μM. TBBPS and the three derivatives with phenol groups, TBBPS-MAE, TBBPS-MBAE and TBBPS-MDBPE, inhabited 50% of the cell viability at a much lower concentration than TBBPS-BAE and TBBPS-BDBPE.

## Discussion

Since the standards of TBBPS-BDBPE byproducts were not commercially available, TBBPS-MAE, TBBPS-MDBPE and TBBPS-MBAE were self-synthesized and further characterized by ^1^HNMR, UHPLC-Orbitrap Fusion HRMS (Thermo Fisher scientific, USA) and HPLC-UV. The results indicated the successful synthesis with high purity (>96%) of TBBPS-MAE, TBBPS-MBAE and TBBPS-MDBPE. These compounds could be used as the standards for the further analysis.

In order to identify the target compounds in samples, an accurate and sensitive method was developed. By using the highly sensitive Orbitrap Fusion HRMS, the IDLs for TBBPS-MAE, TBBPS-MBAE and TBBPS-MDBPE were in the range 0.06–0.1 pg which were lower than those acquired with HPLC-ESI-MS/MS. The target compounds could be identified according to the accurate m/z values of precursor ions within a mass tolerance of 5 ppm. Meanwhile, in the full scan mode, the isotope information was positively observed with the quantification process. As for optimizing the extraction method, the ultrasound method showed slightly higher recoveries and lower SDs which meant it was a stable and reliable extraction method. Meanwhile, the matrix effects were all close to 1.0 which indicated the interference from the matrix could be ignored. ENVI-Carb SPE cartridges could effectively eliminate the interference and concentrate the target compounds. The pretreatment method was reliable and repeatable for the identification of TBBPS-MAE, TBBPS-MBAE and TBBPS-MDBPE in mollusk samples. Meanwhile, ISDBP was selected as an appropriate internal standard for the recovery monitor of TBBPS-MAE, TBBPS-MBAE and TBBPS-MDBPE from the mollusk samples. The recoveries of ISDBP were all higher than 80% in all the samples, indicated the recoveries of target compounds from real samples were reliable.

With the proposed method, the existence of TBBPS-MAE, TBBPS-MBAE and TBBPS-MDBPE in technical TBBPS-BDBPE and mollusk samples from the Chinese Bohai Sea was studied in detail. In the sampling area of this work, several BFRs factories produce TBBPS and TBBPS-BDBPE on a large scale. TBBPS-MAE, TBBPS-MBAE and TBBPS-MDBPE were detectable in technical TBBPS-BDBPE of the BFRs factory with the concentrations ranged from 28 to 394 μg/g. Consequently, in mollusk samples, TBBPS-MAE, TBBPS-MBAE and TBBPS-MDBPE were detectable at a level of ng/g dw. Therefore, the BFRs factories might be the point sources of TBBPS-MAE, TBBPS-MBAE and TBBPS-MDBPE. They were probably produced with the manufacture process, and leaked into the environment through the production and application process. The production process of TBBPS-BDBPE might influence the concentration and detection frequency of the byproducts. TBBPS-BDBPE is synthesized from TBBPS-BAE, and TBBPS-BAE is synthesized from TBBPS. Therefore, TBBPS-MDBPE, which has the most similar structure with TBBPS-BDBPE, is the main byproduct of TBBPS-BDBPE. As a result, TBBPS-MDBPE was detected at the highest concentration and detection frequency. While the technical TBBPS-BAE is produced as intermediate of TBBPS-BDBPE, its structure related byproduct, TBBPS-MAE, showed the lowest concentration and detection frequency. TBBPS-MAE and TBBPS-MBAE were detected in the mollusk samples at the similar concentration level with previously reported for TBBPA derivatives, TBBPA-MAE and TBBPA-MDBPE^3^. TBBPS-MDBPE showed higher concentration level (>1.0 ng/g dw) than TBBPA-MDBPE (<1.0 ng/g dw) in the mollusk samples.^3^ The detected concentrations of TBBPS-MAE, TBBPS-MBAE and TBBPS-MDBPE were lower than Tris-(2,3-dibromopropyl) isocyanurate (TBC)^29^, hexabromocyclododecane (HBCD)^29^ and polybrominated diphenyl ether (PBDE)^29^,^30^ which were also detected in the mollusk samples with the concentration ranges of below detection limit (nd) to 12.1 ng/g dw, nd to 28.8 ng/g dw and 0.01 to 59 ng/g dw, respectively. The detection frequency of TBBPS-MDBPE (95%) was comparable with that reported for HBCD (99%) and PBDE (100%)^29^,^30^. The difference of the concentrations of TBBPS-MAE, TBBPS-MBAE and TBBPS-MDBPE between years was not significant. Interestingly, the detection frequency of TBBPS-MDBPE was 95% which indicated it was probably one widely dispersed brominated compound. Significant difference was not observed among the concentrations of TBBPS-MAE, TBBPS-MBAE and TBBP-MDBPE in different mollusk species. The concentrations of TBBPS-MAE, TBBPS-MBAE and TBBPS-MDBPE remained at a similar level which indicated these compounds could persistently accumulate in the mollusks. The property of persistent accumulation in the biota system may result in their potential health risks posing on the aquatic ecosystem.

Furthermore, the environmental risks of TBBPS-MAE, TBBPS-MBAE and TBBPS-MDBPE were evaluated. Their physical-chemical properties were calculated by US EPA EPI suite. The log *K*_ow_ values of TBBPS-MAE, TBBPS-MBAE and TBBPS-MDBPE were close to TBBPA (7.20)^20^, TBC (7.37)^22^ and HBCD (7.74)^29^ and higher than 5. These results indicated the accumulation of TBBPS-MAE, TBBPS-MBAE and TBBPS-MDBPE in organic materials such as fat-rich organisms. The high *K*_oa_ values of TBBPS-MAE, TBBPS-MBAE and TBBPS-MDBPE indicated low respiratory elimination rate and high bioaccumulation ability in respiratory organisms. Meanwhile, the low *K*_aw_ values implied that large amount of TBBPS-MAE, TBBPS-MBAE and TBBPS-MDBPE would participate in water rather than the air at the boundary exchange process. The comparable *K*_oc_ values with TBBPA (5.24) and TBC (4.92) indicated TBBPS-MAE, TBBPS-MBAE and TBBPS-MDBPE could be absorbed by the sediment and soil with a considerable amount. Usually, the chemicals with log *K*_ow_ > 5 and BCF > 5000 are considered to be bioaccumulative^31^. In addition, the BCF values of TBBPS-MAE, TBBPS-MBAE and TBBPS-MDBPE were higher than TBBPS-BDBPE, TBBPA derivatives and other environmental contaminants in Table 3. They were more bioaccumulative than TBBPS-BDBPE and TBBPA derivatives. As novel brominated contaminants, the toxicity of TBBPS-MAE, TBBPS-MBAE and TBBPS-MDBPE were essential for their environment risks assessment. By using the CGCs as a model, the IC_50_ of TBBPS-MAE, TBBPS-MBAE and TBBPS-MDBPE were all lower than 0.2 μM, suggesting that they were more toxic than TBBPS-BDBPE and TBBPS-BAE with IC_50_ higher than 11 μM (Figure S3). This is probably accused by the phenol group in the structure which probably increased the toxicity of the BFRs^32^. The production of technical products would bring some novel compounds with more severe toxicity into the environment. These byproducts in the environment showed potential health risk to human. Further studies about the toxicity of these byproducts are urgently needed.

**Figure 4:**
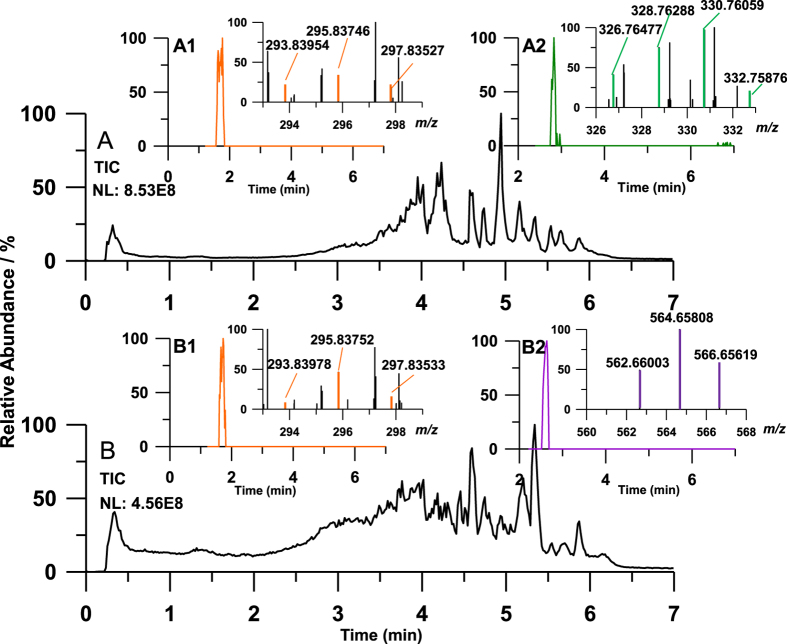
Untargeted screening results of RapL (**A**) and RapS (**B**) sample of 2010. **A1**, **A2**, **B1**, and **B2** were the extracted ion chromatograms (EIC) and mass spectra. A1: NL, 1.33E4, RT, 1.76 min, m/z, 295.83681-295.83977; A2: NL, 6.24E4, RT, 2.82 min, m/z, 328.76215-328.76543; B1: NL, 4.79E4, RT, 1.73 min, m/z, 295.83681-295.83977; B2: NL, 7.08E4, RT, 2.64 min, m/z, 564.65784-564.66348.

By using Orbitrap HRMS, we also found several unknown brominated compounds. With the accurate results determined by Orbitrap HRMS, the compound contained Br is easier to be identified because of the special properties: 1) m/z value of the decimal part decreases with the number increase of ^81^Br; 2) the isotope ratio is different for compounds contained different number of Br. In this study, some untargeted brominated organics also showed up in the samples. Their spectra are shown in Fig. 4 and the molecular formulas were calculated by Xcalibur software. The three compounds detected in mollusk samples showed the properties of containing bromine atoms. Within delta ppm <5, the m/z detected at RTs 2.8 min (isotope ratio, 1:3:3:1), 2.6 min (isotope ratio, 4:6:4) and 1.7 min (isotope ratio, 1:2:1) were calculated to be [C_6_H_2_OBr_3_]^−^, [C_12_H_5_O_4_Br_4_S]^−^ and [C_6_H_2_O_3_NBr_2_]^−^, respectively. They might be three kinds of bromophenols. These three untargeted compounds were further analyzed with Orbitrap Fusion HRMS with reference to the standards, including TBBPS, 2,6-dibromo-4-nitrophenol (DBNP) and four kinds of tribromophenol (Figure S4). The untargeted peak detected at RT 2.64 min (Fig. 4B2) showed similar RT and mass spectra with TBBPS (RT 2.62 min, isotope ratio 1:4:6:4:1). The untargeted peak detected at RT 1.76/1.73 min (Fig. 4A1) had the similar RT and mass spectra with DBNP (RT 1.72 min, isotope ratio 1:3:1). DBNP was also identified as novel bromophenol compounds showing toxicity and potential risk to human^33^,^34^,^35^. DBNP could formed in the chlorination of drinking water and saline sewage effluent^36^. The untargeted peak detected at RT 2.82 min (Fig. 4A2) presented the similar mass spectra with all the four kinds of tribromophenol and the similar RTs with 2,3,4-tribromophenol, 2,4,6-tribromophenol and 2,4,5-tribromophenol. It might be 2,4,6-tribromophenol as it is one kind of mass-produced BFRs in the sampling area. We did not quantify these untargeted compounds because of the lack of reliable pretreatment method. However, their presence in the mollusk samples could be determined with the quantification of our target compounds by Orbitrap Fusion HRMS. In all the 38 mollusk samples, 7 samples were detected to contain tribromophenol, 8 samples for TBBPS and 17 samples for DBNP. The detection frequencies of these three compounds were all higher than TBBPS-MAE. The anthropogenic activities might result in the emergence of 2,4,6-tribromophenol, DBNP and TBBPS in the environment as they were not reported as natural compounds^37^. The untargeted compounds might also become novel brominated contaminants. In this view, further investigation is needed to be conducted on the identification and environmental fate of these compounds. It is worth mentioning that the Orbitrap Fusion HRMS is a powerful tool for the quantification of novel contaminants and qualitative analysis of unknown contaminants with one injection.

Most BFRs, such as poly brominated diphenyl ether (PBDE), TBBPA and TBBPS derivatives, usually share the ether bond linked structure^6^. For the production of ether bond derived organic aromatic chemicals with several bromine atoms, the left-over starting reagents, co-produced phenol and less brominated byproducts could be potential environmental contaminants together with the desired BFRs products. The byproducts generated from manufacture production or degradation draw great attention because they were found in various environment compartments as novel or emerging BFRs^38^,^39^,^40^,^41^. For example, the byproducts of TBBPA and its derivatives, TBBPA-MAE, TBBPA-MDBPE, TBBPA mono(2-hydroxylethyl ether), TBBPA mono(glycidyl ether), dibromobisphenol A and tribromobisphenol A have been determined in water, soil and biota system^2^,^3^,^42^. In this work, we found the manufacture process of TBBPS-BDBPE resulted in the occurrence of TBBPS-MAE, TBBPS-MBAE and TBBPS-MDBPE inevitably. The existence of the phenol byproducts in the aryl-O linked technical products might be a global problem. Although aryl-O bond of organic chemicals is considered very stable in chemical reactions, its cleavage is easy to fulfill under the bacterial biodegradation, the UV irradiation and super-reduced conditions^43^,^44^,^45^. TBBPA bis(2,3-dibromopropyl ether) (TBBPA-BDBPE) was also found to transform to TBBPA via ether breakage in aquatic mesocosm^46^. The compounds with ether bond are not as stable as suppositional under environmental conditions. TBBPA-MAE and TBBPA-MDBPE were also predicted to be the degradation products of TBBPA bis(allyl ether) (TBBPA-BAE) and TBBPA-BDBPE by the University of Minnesota Pathway Prediction System^2^,^3^. Through the same microbial transformation, TBBPS derivatives showed the potential ability of ether bond cleavage and form the mono-modified degradation products, TBBPS-MAE and TBBPS-MDBPE. The co-produced byproducts in manufacture process and microbial degradation in the environment contribute to the occurrence of mono-modified byproducts in the environment. The study about the byproducts and degradation products of these ether linked BFRs will supplement the information for novel brominated contaminants.

In conclusion, TBBPS-MAE, TBBPS-MBAE and TBBPS-MDBPE were identified as three novel brominated contaminants, which showed higher bioaccumulation properties and potential severe toxicity compared with TBBPS-BDBPE. They could be co-produced and leaked into the environment along with production process of TBBPS-BDBPE. The occurrence of the mono-modified byproducts or degradation products of the extensively used brominated products might be a widespread problem. This work could promote the further study of the environmental fate and risks of widely used TBBPS and TBBPS-BDBPE. The strategy used in this work, integrating the synthesis of standards and Orbitrap HRMS identification, could also be an effective approach for identifying other novel brominated pollutants related to BFRs.

## Methods

### Chemicals and Materials

TBBPS (98%) was purchased from Beijing Apisi biotechnology co. ltd., and was used without further purification. Ammonium hydroxide (50%) was purchased from Sigma-Aldrich. Methanol, acetone, hexane and methylene dichloride (DCM) were all HPLC grade. Ultra-pure water was generated by a Milli-Q advantage A10 system. TBBPS-MAE, TBBPS-MBAE and TBBPS-MDBPE were synthesized and purified in our lab. The synthesis procedures were described in Supporting Information. The purities of these three compounds were 99%, 98% and 96% as determined by HPLC-UV (214 nm).

### Sample Collection

From 2009 to 2013, in August of each year, 11 species of mollusks were collected from one coastal city — Shouguang, Shandong Province. These 11 selected species of mollusks were Rapana venosa (large and small, RapL and RapS), Crassostrea talienwhanensis (Ost), Scapharca subcrenata (Sca), Cyclina sinensis (Cyc), Mya arenaria (Mya), Mactra veneriformis (Mac), Chlamys farreri (Chl), Neverita didyma (Nev) and Meretix meretrix (large and small, MerL and MerS) (Fig. 3, Table S1). After sampling, the mollusks were frozen and transported on ice to the laboratory, and then cleaned by water. The collected samples were disposed according to the previous method^29^. The samples were freeze-dried, grinded, and preserved at −20 °C until analysis. A total of 38 mollusk samples were obtained and analyzed.

### Sample Pretreatment

#### Ultrasound Extraction

Mollusk (0.5 g) samples were mixed with 2 g anhydrous Na_2_SO_4_; spiked with 10 ng ^13^C labeled 3,5-dibromophenol (ISDBP); extracted with 10 mL DCM/hexane (8/2, V/V) for three times by sonication (30 minutes per time). After centrifugation, the extraction solution was collected and the solvent was removed with rotary evaporator and re-dissolved in 3 mL DCM/hexane (1/1, V/V) before SPE process.

#### SPE Procedures

The SPE cartridges (Supelclean^TM^ ENVI-Carb^TM^, 0.5 g, 6 mL) were first conditioned by 5 mL acetone, 5 mL DCM and 10 mL hexane and then the samples were loaded. Then the cartridges were cleaned by 5 mL hexane and 5 mL DCM/hexane (1/1, V/V). Finally, the cartridges were eluted with 10 mL acetone (containing 0.5% NH_3_·H_2_O) and the elution were collected and blown to dryness by gentle nitrogen gas flow. The residue was solvent-exchanged to 1 mL methanol and analyzed by UHPLC-Orbitrap Fusion HRMS.

### Instrument Parameters for UHPLC-Orbitrap Fusion HRMS, HPLC-UV and HPLC-ESI-MS/MS Analysis

The details were described in Supporting Information.

### Analytical Method Validation

TBBPS-MAE, TBBPS-MBAE and TBBPS-MDBPE in samples were identified by retention time and accurate m/z of the precursor ions comparison with the corresponding standards. Quantification of the target compounds in the environmental samples was performed by peak area of the accurate precursor ions of compounds within 5 ppm mass tolerance. For TBBPS-MAE, TBBPS-MBAE and TBBPS-MDBPE, precursor ions at m/z 604.69189, 682.60248 and 764.52643 were used as quantification ions, and m/z 602.69415, 684.60022 and 762.52856 were used as the qualitative ions.

All calibration standards and spiking solutions were prepared by serial dilution in methanol. A linear calibration curve with seven points ranged from 0.05 to 100 ng/mL was used to quantify the target compounds with a determination coefficient (*R*^2^) higher than 0.99 (Table 2). The concentrations of target compounds were determined by an external standard method. Every 9 samples were prepared with one blank sample (only anhydrous Na_2_SO_4_ added), and analyzed with methanol as solvent blank to make sure no cross contamination. The DLs were determined by the lowest mass value of the target compounds that Orbitrap Fusion HRMS detected. The IDLs were determined for five times within 20% relative standard deviation for the signals. The MDLs were based on replicate analysis (n = 10) of Nev sample spiked at a mass concentration of 5 times of the IDLs and calculated with the method previously used for HRMS^47^. The recoveries were determined at the spiking amounts of 1 ng (n = 5), 10 ng (n = 7) and 100 ng (n = 5) in 0.5 g Nev samples (not containing target compounds). The internal standard ^13^C labeled 3,5-dibromophenol (ISDBP) was used to monitor the pretreatment process and not used for the concentration calculation. The detailed procedures for the SPE optimization and results were described in Supporting Information. The matrix effects were determined according the method reported elsewhere previously^43^,^48^. Detailed information regarding the synthesis routines and ^1^HNMR and MS^2^ spectra of TBBPS-MAE, TBBPS-MBAE and TBBPS-MDBPE, the ^1^HNMR data, instrumental analysis information of Orbitrap Fusion HRMS, HPLC-UV and HPLC-ESI-MS/MS, the optimization of pretreatment method, the concentration of every mollusk sample, the cell information and cytotoxicity test method, the cytotoxicity of TBBPS and its derivatives, the HRMS chromatograms and spectra of different brominated phenols are provided in the Supporting Information.

## Additional Information

**How to cite this article**: Liu, A.-f. *et al*. Characterization of Three Tetrabromobisphenol-S Derivatives in Mollusks from Chinese Bohai Sea: A Strategy for Novel Brominated Contaminants Identification. *Sci. Rep*. **5**, 11741; doi: 10.1038/srep11741 (2015).

## Supplementary Material

Supplementary Information

## Figures and Tables

**Table 1:** The HRMS information of synthesized TBBPS-MAE, TBBPS-MBAE and TBBPS-MDBPE.

	TBBPS-MAE	TBBPS-MBAE	TBBPS-MDBPE
Detected [M-H]^−^ Ions (m/z)	604.69189	684.60022/682.60248	764.52643
Calculated [M-H]^−^ Ions (m/z)	604.69196	684.60043/682.60247	764.52659
Mass Error (ppm)	0.12	0.31/0.01	0.21
Calculated Molecular Formula	[C_15_H_9_Br_4_O_4_S]^−^	[C_15_H_8_Br_5_O_4_S]^−^	[C_15_H_9_Br_6_O_4_S]^−^
Detected Isotopic [M-H]^−^ Ions (m/z)	600.69635/602.69415/604.69189/606.68958/608.68701	678.60681/680.60474/682.60248/684.60022/686.59790/688.59528	758.53296/760.53082/762.52856/764.52643/766.52423/768.52185/770.51947
Isotope Distribution	1:4:6:4:1	1:5:10:10:5:1	1:6:15:20:15:6:1

**Table 2:** The recoveries and matrix effects (MEs) of TBBPS-MAE, TBBPS-MBAE, TBBPS-MDBPE and internal standard from spiked mollusk samples (0.5 g Nev sample).

	Calibration Curve(0.05–100 ng/mL)	*R*^*2*^	ASERecoveries10 ng (n = 7)	Ultrasound Recoveries			MEs (n = 3)			
				1 ng (n = 5)	10 ng (n = 7)	100 ng (n = 5)	1 ng/mL	5 ng/mL	10 ng/mL	
TBBPS-MAE	Y = 304964 + 519149*X	0.9907	86 ± 13%	78 ± 9%	85 ± 7%	85 ± 4%	1.05 ± 0.05	0.92 ± 0.08	0.86 ± 0.08	
TBBPS-MBAE	Y** **=** **191635 + 421073*X	0.9955	85 ± 11%	70 ± 4%	90 ± 10%	75 ± 5%	0.90 ± 0.11	0.90 ± 0.12	0.89 ± 0.11	
TBBPS-MDBPE	Y** **=** **73875 + 363618*X	0.9972	76 ± 14%	92 ± 8%	80 ± 10%	71 ± 9%	1.03 ± 0.17	0.94 ± 0.05	0.91 ± 0.07	
ISDBP			76 ± 10%		101 ± 7%		0.92 ± 0.06	1.03 ± 0.05	0.89 ± 0.07	

**Table 3:** Physical-chemical constants a of TBBPS, TBBPS derivatives and other BFRs with environmental significance.

	WS^b^	log *K*_ow_^c^	log *K*_aw_^d^	log *K*_oa_^e^	log *K*_oc_^f^	BCF^g^
TBBPS-MAE	1.26E-03	6.61	−11.68	18.29	4.83	10730
TBBPS-MBAE	1.75E-04	7.01	−12.38	19.39	5.05	13200
TBBPS-MDBPE	2.50E-05	7.36	−13.43	20.79	5.24	8829
TBBPS-BAE	1.13E-05	8.02	−8.81	16.83	5.50	4207
TBBPS-BDBPE	4.18E-09	9.52	−12.31	21.83	6.33	775
TBBPS	3.66E-02	5.21	−14.56	19.77	4.16	1266
TBBPA-MAE	3.46E-05	8.61	−8.15	16.76	5.91	2163
TBBPA-MDBPE	6.91E-07	9.36	−9.90	19.26	6.33	928
TBBPA-BAE	3.12E-07	10.02	−5.28	15.30	6.58	442
TBBPA-BDBPE	1.16E-10	11.52	−8.78	20.30	7.41	81
TBBPA	1.00E-03	7.20	−11.03	18.23	5.24	10580
TBP246	9.13	4.18	−5.84	9.97	3.38	247
DBNP	9.07	3.69	−4.34	8.03	3.35	126
TBC	1.14E-05	7.37	−16.31	23.68	4.92	8732
BPAF	4.30	4.47	−7.63	12.10	3.73	416
BPA	172.7	3.64	−9.11	12.75	3.10	72
HBCD	2.09E-05	7.74	−4.15	11.89	6.72	5759

DBNP, 2,6-dibromo-4-nitrophenol; BPAF, bisphenol AF; TBC, tris-(2,3-dibromopropyl) isocyanurate; BPA, bisphenol A; HBCD, hexabromocyclododecane.

^a^Calculated by US EPA Suite V 4.1;

^b^Water solubility, (mg/L);

^c^octanol-water partition coefficient;

^d^air-water partition coefficient;

^e^octanol-air partition coefficient;

^f^soil absorption coefficient;

^g^bioconcentration factor (L/kg, wet-wt); TBP246, 2,4,6-tribromophenol.
