# Supplementary material for: Characterization of Three Tetrabromobisphenol-S Derivatives in Mollusks from Chinese Bohai Sea: A Strategy for Novel Brominated Contaminants Identification
Source: Sci Rep. 2015 Jul 1;5:11741. doi: 10.1038/srep11741 (PMC4486981; doi:10.1038/srep11741)
Supplement: Supplementary Information [file srep11741-s1.pdf]

Supporting Information for

Characterization of Three Tetrabromobisphenol-S Derivatives in Mollusks from Chinese Bohai Sea: A Strategy for Novel Brominated Contaminants Identification

Ai-feng Liu, Yong Tian, Nuo-ya Yin, Miao Yu, Guang-bo Qu, Jian-bo Shi*, Yu-guo Du, Gui-bin Jiang

State Key Laboratory of Environmental Chemistry and Ecotoxicology, Research Center for Eco-Environmental Sciences, Chinese Academy of Sciences, P.O. Box 2871, Beijing 100085, China

* Corresponding author

Tel/fax: +86-10-62849129

E-mail: jbshi@rcees.ac.cn (Jianbo Shi)

Number of tables: 1

Number of pages: 12

Number of Figures: 4

^{1}HNMR Data

TBBPS mono(allyl ether) (TBBPS-MAE)

^{1}HNMR (400 MHz, DMSO) δ 8.07 (s, 2 H), 7.72 (s, 2 H), 6.14-6.06 (m, 1 H), 5.45-5.44 (d, *J*=1.6 Hz, 1 H), 5.41-5.40 (d, *J*=1.6 Hz, 1 H), 4.55-4.53(d, *J*=5.6 Hz, 2 H).

TBBPS mono(2-bromoallyl ether) (TBBPS-MBAE)

^{1}HNMR (400 MHz, DMSO) δ 8.09 (s, 2 H), 7.7 (s, 2 H), 6.23 (s, 1 H), 5.81 (d, *J*=1.6 Hz, 1 H), 4.66(s, 2 H).

TBBPS mono(2,3-dibromopropyl ether) (TBBPS-MDBPE)

^{1}HNMR (400MHz, DMSO) δ 8.36 (s, 2 H), 8.26 (s, 2 H), 4.75-4.73 (t, *J*=10.8, 5.6 Hz, 1 H), 4.45-4.36(m, 2 H), 4.12-4.07 (t, *J*=17.2, 6.0 Hz, 2 H).

Synthesis Procedures of TBBPS-MAE, TBBPS-MDBPE and TBBPS-MBAE

TBBPS-MAE To a solution of TBBPS (1.98 g) and allyl bromide (0.3 ml) in acetone (10 mL), Sodium hydroxide (0.28 g in 2 mL water) was added at room temperature. The reaction mixture was stirred for 5 hr. After the thin-layer chromatography (TLC) showed the disappearance of the reagent, the reaction mixture was diluted with CH_2Cl_2 and washed with saturated solution of sodium bicarbonate and water. The organic layer was dried over $MgSO_4$. After removal of the organic solvent, the residue was purified by column chromatography, giving TBBPS-MAE in a yield of 48%.

TBBPS-MDBPE To a solution of TBBPS-MAE (1.2 g) in carbon tetrachloride (12 mL), bromine (0.205 ml) was added at room temperature. The reaction mixture was stirred for 1 hr. After the TLC showed the disappearance of TBBPS-MAE, the reaction mixture was diluted with CH_2Cl_2 and washed with saturated sodium

thiosulfate, saturated sodium bicarbonate and water. The organic layer was dried over $MgSO_4$. After removal of the organic solvent, the residue was purified by column chromatography, giving TBBPS-MDBPE in a yield of 87%.

TBBPS-MBAE To a solution of TBBPS-MDBPE (766 mg) in acetonitrile (20 ml), 25 mL KOH (5 mol/L) was added, the reaction mixture was heated and reflex for 2 hrs, and then cooled to room temperature. The mixture was extracted with DCM and purified by column chromatography, giving TBBPS-MBAE in a yield of 47%.

Instrument Analysis

UHPLC-Orbitrap Fusion HRMS A Thermo Ultimate 3000 UHPLC coupled with a Orbitrap Fusion tribrid mass spectrometer (Thermo, USA) and Extend C18 column (2.1×50 mm, 1.8 μm, Agilent) was applied for the analysis of the environmental samples and technical products analysis. Methanol (A) and 0.1% formic acid water (B) were applied as the mobile phases at a flow rate of 0.4 mL/min. The flow gradient was 60:40 (v/v, A/B) initially, and kept for 1 min, then increased to 90% A in 2 min, kept for 2 min and returned to 60:40 (v/v, A/B) in 0.1 min and kept for 1.9 min. Electrospray ionization was used as the ionization method and operated in the negative ion mode. The full-scan ranged from m/z 100 to 1000 and MS^2 mode using higher-energy collisional dissociation (HCD) as fragmentation technique under heated electrospray ionization (HESI) mode. The parameters for the mass spectrometer are summarized as follows: spray voltage, 2500 V; sweep gas flow rate, 1 respective arbitrary units (RAU); sheath gas flow rate, 40 RAU; aux gas flow rate, 10 RAU; ion transfer tube temperature, 350 ℃; vaporizer temperature, 200 ℃; MS^1 detector, Orbitrap; MS^1 resolution, 120,000; MS^1 scan range, 100-1000; MS^1 maximum injection time, 100 ms; MS^1 automated gain control (AGC) target, 100,000; S-lens RF level, 60 V; MS^2 HCD collision energy, 45%; MS^2 detector, Orbitrap; MS^2

resolution, 15,000; MS^2 AGC target, 50,000; MS^2 maximum injection time 35 ms; MS^2 start mass, 50. The data determined by UHPLC-Orbitrap Fusion HRMS were processed with Xcalibur software (Thermo Fisher Scientific, USA). For the quantification of the small molecular compounds, a resolution of 120,000 at normal level of mass, could guarantee both the accuracy and scan speed. The selected m/z of $[M-H]^-$ ion was extracted from the total ion chromatography with a mass tolerance of 5 ppm. The target compounds could be effectively distinguished from the interfering substances with the close m/z value.

HPLC-UV Parameters A Thermo Ultimate 3000 HPLC coupled with a UV detector (Thermo Fisher Scientific, USA) and ZORBAX ODS column (150×3 mm, 5 μm, Agilent) was applied for the purities determination of synthesized compounds. Methanol (A) and 0.1% formic acid water (B) were applied as the mobile phases at a flow rate of 0.35 mL/min. The flow gradient was 60:40 (v/v, A/B) initially, and kept for 1 min, then increased to 100% A in 11 min and then returned to 60:40 (v/v, A/B) in 1 min and kept for 2 min. The wavelength of the detector was set at 254 nm because of the better UV absorption and lower baseline noise.

Waters HPLC-MS/MS Analysis Parameters A Waters 2695 HPLC coupled with a triple-quadrupole mass spectrometer (Quattro Premier XE, Waters, USA) and ZORBAX ODS column (150×3 mm, 5 μm, Agilent) was applied to compare the instrument detection limits (IDLs) with HRMS. For the MS/MS analysis, Methanol (A) and 0.1% formic acid water (B) were applied as the mobile phases at a flow rate of 0.35 mL/min. The flow gradient was 60:40 (v/v, A/B) initially, and kept for 1 min, then increased to 100% A in 11 min and then returned to 60:40 (v/v, A/B) in 1 min and kept for 2 min. Electrospray ionization was used as the ionization method and operated in the negative ion mode. The capillary voltage was 2.5 kV, source

temperature and desolvation temperature were 120 ℃ and 450 ℃, respectively. Desolvation gas and cone gas flow were 400 L/h and 100 L/h, respectively. Argon gas, used for the ionization of daughter ion in the collision cell, was set at a flow rate of 0.25 L/min. MS/MS parameters were optimized in infusion experiments using individual standard solution at a concentration of 100 ng/mL in methanol. The precursor ion was obtained from the mass scan mode with the optimized parameters for cone voltage and capillary voltage. Mass scan mode was run by scanning from m/z 50 to 1000, using a scan time of 0.2 s with a step size of 0.1 amu and a pause of 100 ms between two scans. Fragment ion was optimized by the collision energy to obtain the best ion intensity in daughter scan mode. MRM mode was used for the quantification of target compounds. The optimized cone voltages were -28 V, -20 V, and -25 V for parent ions of TBBPS-MAE (605.0), TBBPS-MBAE (684.8) and TBBPS-MDBPE (764.8), respectively. These three compounds had same quantitative ion (563.9) and qualitative ion (78.9), and the optimized collision energy for quantitative and qualitative ion were -30 and -60 eV (TBBPS-MAE), -32 and -70 eV (TBBPS-MBAE), -42 and -70 eV (TBBPS-MDBPE), respectively.

The Optimization of Pretreatment Method

Accelerated Solvent Extraction Nev samples (0.5 g) samples were mixed with 15g anhydrous Na_2SO_4; spiked with 10 ng ^{13}C labeled 3,5-dibromophenol and 10 ng target compounds; extracted with 100 mL of DCM/hexane (8/2, v/v) at 100 ℃ and 1500 psi using accelerated solvent extraction(ASE 300, Dionex) for 12 min with 3 cycles. Then, the extraction solvent was removed by rotary evaporator and solvent-exchanged with 3 mL of DCM/hexane (1/1) before solid phase extraction process.

SPE Optimization Nev samples (0.5g,) collected in Dalian, Liaoning Province in 2010 were used to evaluate the pretreatment method. Herein, the standards were spiked in 0.5 g samples, and DCM/hexane (8/2) was used as the extraction solvent to compare ASE and ultrasound method for the recovery. Three kinds of SPE cartridges (Supelclean^{TM} ENVI-Carb^{TM}, LC-Florisil$^{@}$, LC-Si, 500 mg, 6 mL) were used to evaluate the clean and concentration efficiency. First of all, the standards were spiked in samples and extracted by different method. The extraction solution was solvent exchanged to 3 mL DCM/hexane (1/1) and loaded onto the cartridges after the cartridges were preconditioned with 5 mL acetone, 5 mL DCM and 5mL hexane. Then the cartridges were eluted with 5 mL hexane, 5mL DCM/hexane (1/1), 5 mL DCM and 10 mL acetone (or 10 mL 0.5% ammonium hydroxide/acetone). All the elution was collected and solvent-exchanged to 1 mL methanol and analyzed by UHPLC-Orbitrap Fusion HRMS. All the three SPE cartridges showed great absorption for the target compounds. The standards could only be eluted by acetone. Meanwhile, we found that 20 mL acetone was better than 10 mL as the elution solvent for the recovery. Because phenols are weak acid, it is helpful for the elution of the target compounds when a slight amount of weak basic was used to assist the elution. It was found that 10 mL 0.5% $NH_3 \cdot H_2O$/acetone could elute the loaded compounds efficiently. The matrix of the samples affected the absorption ability of the cartridges which resulted in the elution of target compounds in DCM. But it was sufficient to eliminate the unwanted compounds from the cartridges with 5 mL hexane and 5mL DCM/hexane (1/1). Finally, the cartridges were washed with 5 mL hexane and 5mL DCM/hexane (1/1), and eluted with 10 mL 0.5% $NH_3 \cdot H_2O$/acetone. The elution from LC-Florisil$^{@}$ and LC-Si cartridges showed obvious yellow color, and this phenomenon was not observed by use of ENVI-Carb^{TM} cartridges. The results

revealed the strong absorption capacity of ENVI-Carb™ cartridges for the interference, especially the pigment. Supelclean™ ENVI-Carb™ cartridge (500 mg, 6 mL) was the best option for the purification treatment and concentration of TBBPS-MAE, TBBPS-MBAE and TBBPS-MDBPE.

The Method of Toxicity Test Used Cerebellum Granule Cells (CGCs)

Cells Information CGCs were prepared from 7 days old SD rat. The cerebella were dissociated in HBSS and plated at 200 μL ($0.5*10^6$ cells/mL) in 96-well plates coated with poly-L-lysine. Cultures were maintained in DMEM-F12 medium with 5% heat inactivated horse serum, 5% heat inactivated fetal bovine serum, 1% L-glutamine, 25 mM KCl, and antibiotics (penicillin 100 U/mL and streptomycin 100 μg/mL). Cells were maintained at 37℃ in a humidified atmosphere of 5% CO_2. Arabinofuranosylcytosine (10 μM) was added to the culture medium after plating for 18-22 hrs to prevent proliferation of non-neuronal cells. The cells were cultured for 7 days until synapses formed.

Cell Viability Using AB Assay To measure the cytotoxicity of TBBPS and derivatives to CGN, various concentrations of compounds (0.01, 0.05, 0.1, 0.25, 0.5, 0.75, 1.0, and 2.0 μg/mL) were added. After exposure for 24 hrs, the cell viability was determined using AB assay. The fluorescence was measured using excitation wavelength of 530 nm and emission wavelength of 590 nm in a multiwell fluometric reader (Thermo, USA).

Table S1. TBBPS-MAE, TBBPS-MBAE and TBBPS-MDBPE detected in mollusks (ng/g dw).

Compound detected	years	mollusks species										
		RapL	RapS	Ost	Sca	MerL	MerS	Mac	Cyc	Mya	Chl	Nev
TBBPS-MAE	2009	<MDL	<MDL	<MDL	0.1				<MDL		<MDL	<MDL
	2010	<MDL	<MDL		<MDL	<MDL	<MDL	<MDL	<MDL	<MDL		
	2011	<MDL	<MDL	<MDL	<MDL	<MDL	<MDL	<MDL			<MDL	
	2012	<MDL	<MDL	<MDL	<MDL		<MDL	<MDL			<MDL	<MDL
	2013	<MDL	<MDL	0.2	<MDL		<MDL	<MDL			<MDL	
TBBPS-MBAE	2009	<MDL	0.5	0.3	0.3				0.2		0.1	<MDL
	2010	<MDL	0.1		0.1	0.1	<MDL	<MDL	<MDL	0.1		
	2011	<MDL	<MDL	0.3	0.1	<MDL	0.1	<MDL			<MDL	
	2012	<MDL	<MDL	<MDL	<MDL		<MDL	<MDL			<MDL	<MDL
	2013	<MDL	<MDL	1.6	0.1		0.3	<MDL			<MDL	
TBBPS-MDBPE	2009	4.1	1.7	<MDL	1.4				1.0		0.7	0.9
	2010	1.5	2.7		0.6	0.6	1.5	0.4	1.1	0.7		
	2011	1.7	2.9	1.3	1.1	1.4	<MDL	1.2			0.3	
	2012	1.6	3.6	1.7	0.9		0.6	0.9			0.5	0.7
	2013	0.8	1.5	2.5	0.6		0.7	0.9			1.7	

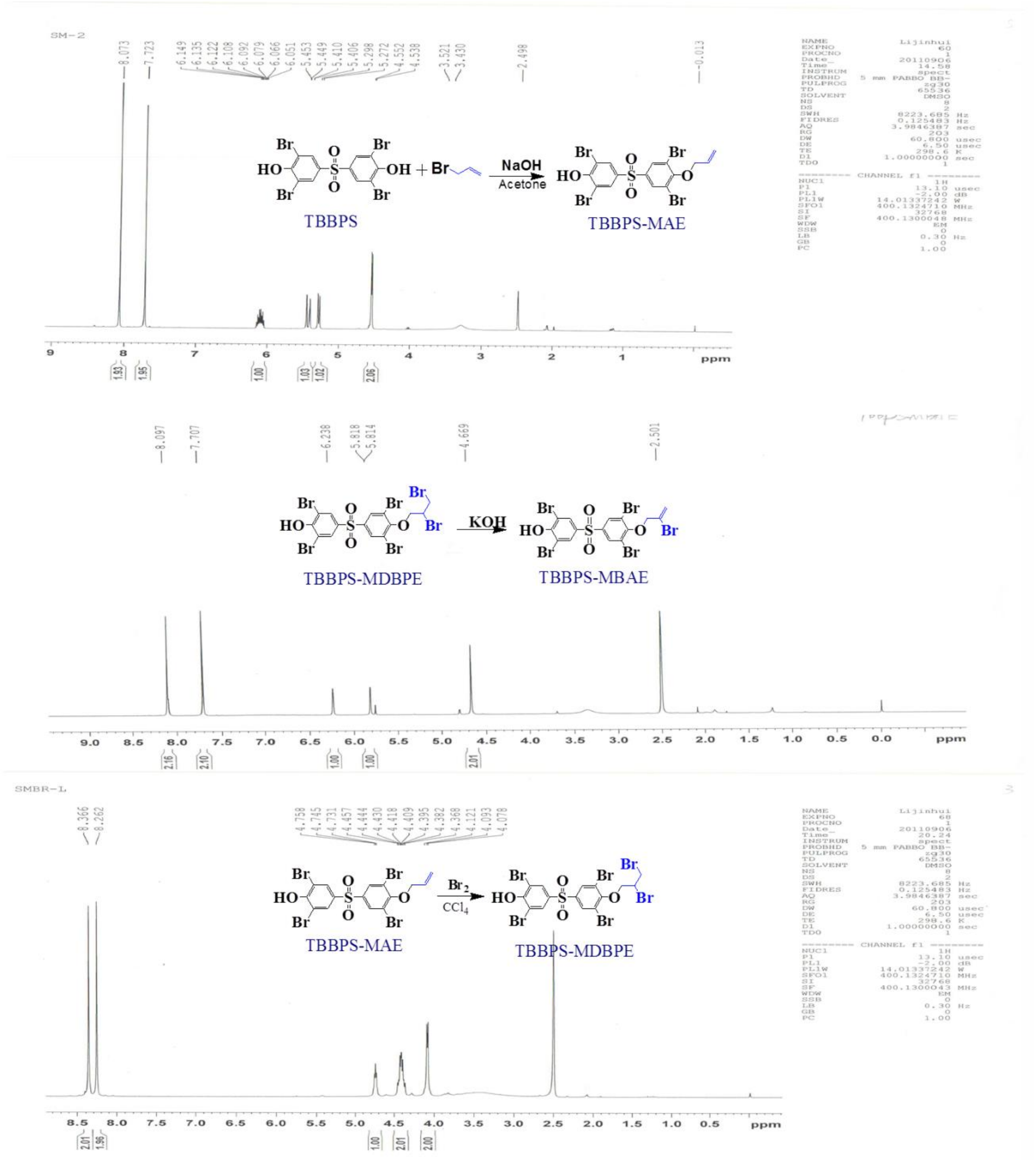

Figure S1. The synthesis routines and ^{1}HNMR spectra of TBBPS-MAE, TBBPS-MBAE and TBBPS-MDBPE.

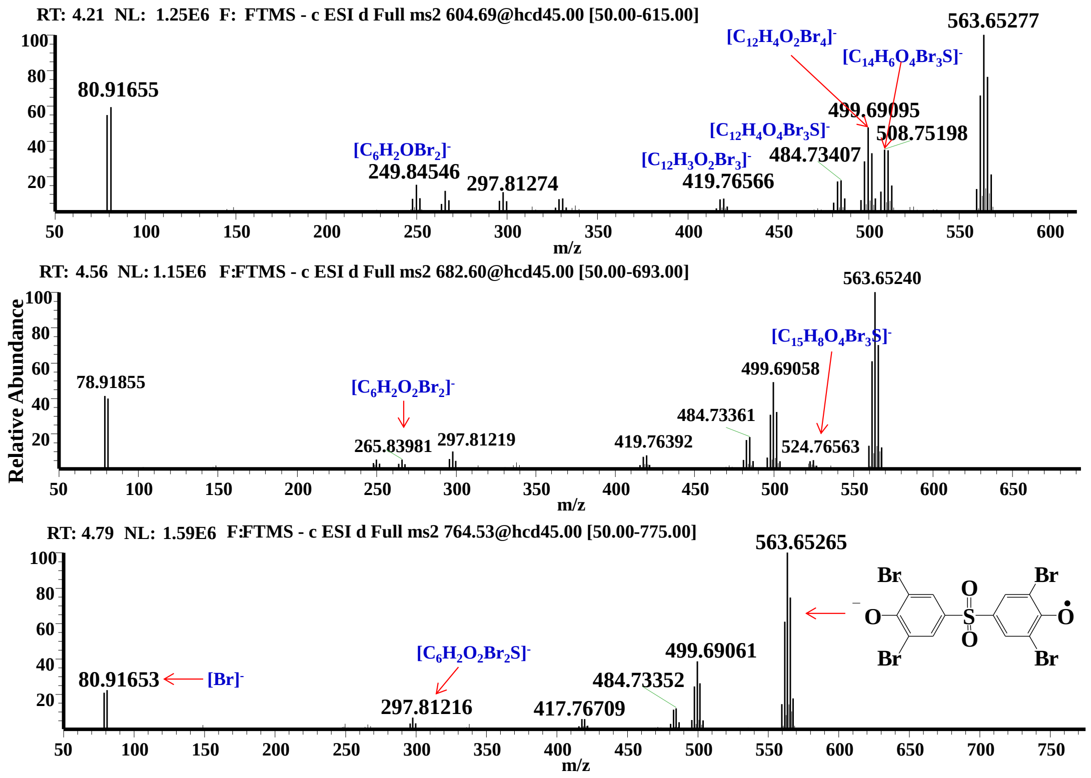

Figure S2. The MS^2 spectra of TBBPS-MAE, TBBPS-MBAE and TBBPS-MDBPE.

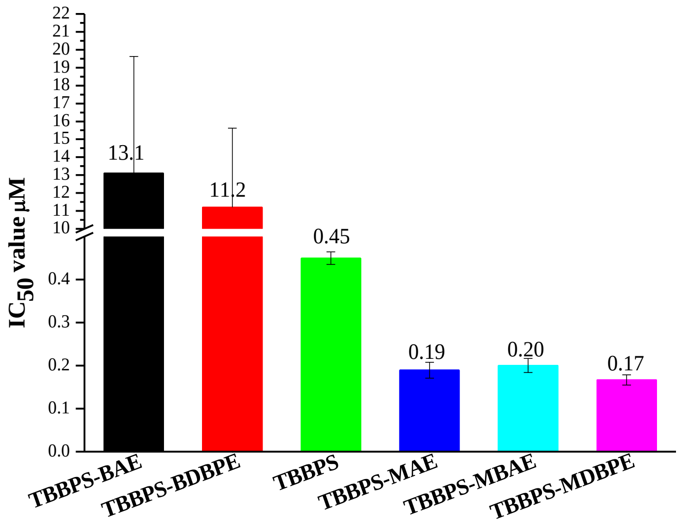

Figure S3. The IC_{50} values of TBBPS, derivatives and impurities acted on CGC cells.

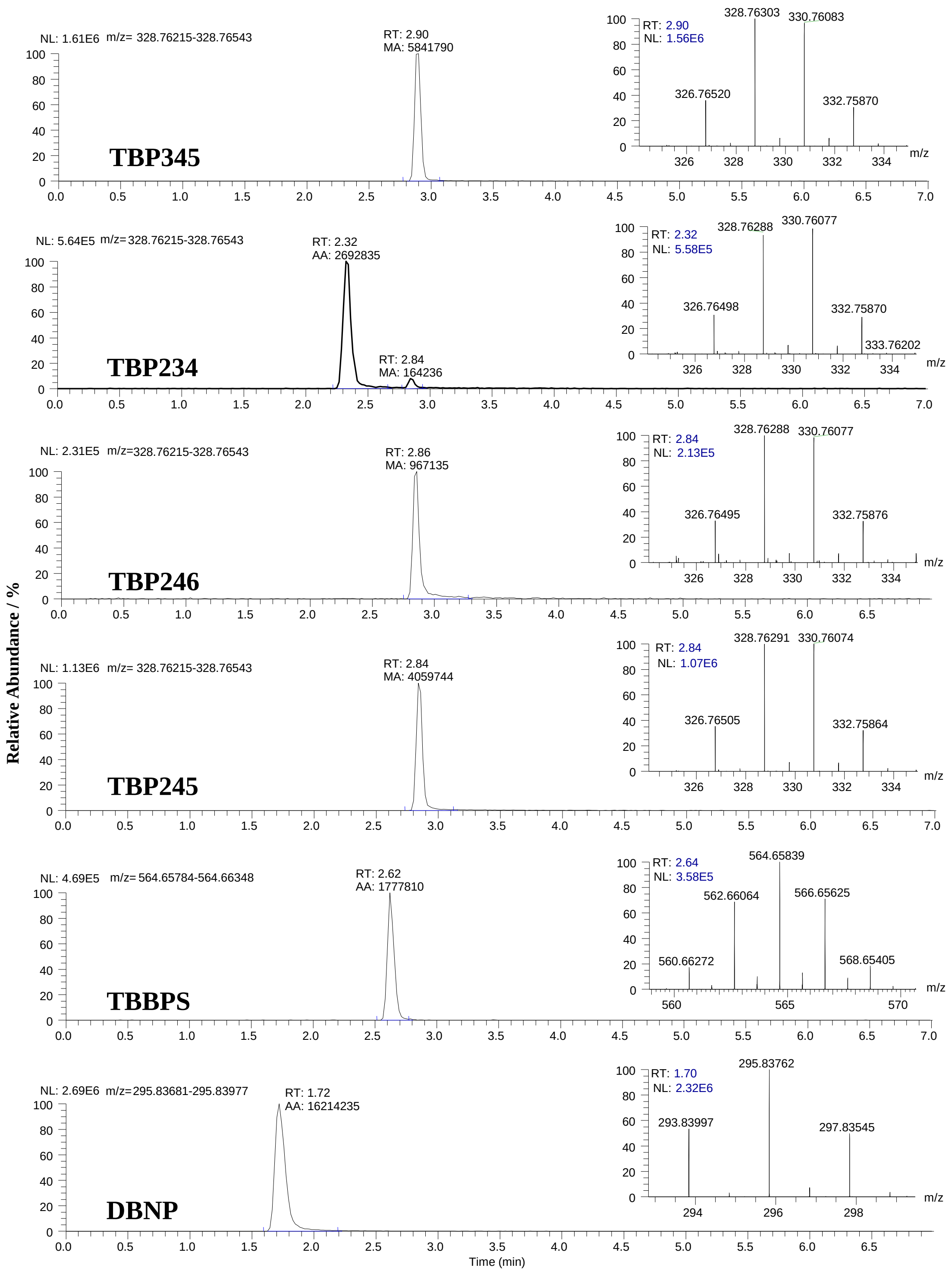

Figure S4. The mass chromatograms and spectra of bromophenols. TBP345, 3,4,5-tribomophenol; TBP234, 2,3,4-tribomophenol; TBP246, 2,4,6-tribomophenol; TBP245, 2,4,5-tribomophenol; TBBPS, tetrabromobisphenol-S; DBNP, 2,6-dibromo-4-nitrophenol.
